# Mechanisms of lung fibrosis induced by carbon nanotubes: towards an Adverse Outcome Pathway (AOP)

**DOI:** 10.1186/s12989-016-0123-y

**Published:** 2016-02-29

**Authors:** Giulia Vietti, Dominique Lison, Sybille van den Brule

**Affiliations:** Louvain centre for Toxicology and Applied Pharmacology, Université Catholique de Louvain, Avenue E. Mounier, 52 - bte B1.52.12, 1200 Brussels, Belgium

**Keywords:** Carbon nanotube, Lung fibrosis, AOP, Fibroblast, Macrophage, Epithelial cell, Mechanisms

## Abstract

Several experimental studies have shown that carbon nanotubes (CNT) can induce respiratory effects, including lung fibrosis. The cellular and molecular events through which these effects develop are, however, not clearly elucidated. The purpose of the present review was to analyze the key events involved in the lung fibrotic reaction induced by CNT and to assess their relationships. We thus address current knowledge and gaps with a view to draft an Adverse Outcome Pathway (AOP) concerning the fibrotic potential of CNT.

As for many inhaled particles, CNT can indirectly activate fibroblasts through the release of pro-inflammatory (IL-1β) and pro-fibrotic (PDGF and TGF-β) mediators by inflammatory cells (macrophages and epithelial cells) via the induction of oxidative stress, inflammasome or NF-kB. We also highlight here direct effects of CNT on fibroblasts, which appear as a new mode of toxicity relatively specific for CNT. Direct effects of CNT on fibroblasts include the induction of fibroblast proliferation, differentiation and collagen production via ERK 1/2 or Smad signaling. We also point out the physico-chemical properties of CNT important for their toxicity and the relationship between in vitro and in vivo effects. This knowledge provides evidence to draft an AOP for the fibrogenic activity of CNT, which allows developing simple in vitro models contributing to predict the CNT effects in lung fibrosis, and risk assessment tools for regulatory decision.

## Background

Carbon nanotubes (CNT) are cylinders with a diameter in the order of nanometers, constituted of one or several graphene layers named single walled (SW) or multi walled (MW) CNT, respectively. They attract wide interests because of their unique physico-chemical properties useful for a variety of applications. In the last few years, several studies have suggested diverse applications of CNT in consumer and industrial products, in fields such as electronics, structural engineering, manufacturing and medicine. Thus, determining the health hazards of CNT is of great importance in view of the increased potential for human exposure within occupational, environmental, medical and consumer environments. In particular, there is a great focus on inhalation exposure because the fibrous shape of CNT, similar to asbestos fibers, and their high surface area raise serious concerns for harmful respiratory effects [1]. Recent experimental studies have indicated that CNT can induce respiratory toxicity, including inflammatory and fibrotic reactions, and peritoneal mesothelioma in animals [2–7]. The National Institute for Occupational Safety and Health (NIOSH) has recommended an occupational exposure limit (REL) of 1 μg/m^3^ together with other strategies for controlling workplace exposures to CNT and for implementing a medical surveillance program of exposed workers [8].

Table 1, 2, 3 shows that CNT can induce fibrotic lung reactions, whatever the mode of experimental administration (inhalation, aspiration or injection) or the animal species used. Depending on their physicochemical characteristics, the fibrogenic activity of CNT appear, however, to vary considerably, pointing out the necessity to identify which CNT property(ies) drive(s) their hazardous activities, and which mechanisms are involved. It is, therefore, important to study and investigate how CNT can exert their pro-fibrotic activities and to initiate prevention strategies to avoid the development of diseases in humans like asbestosis.

**Table 1:** In vivo lung fibrotic reaction of CNT administered via inhalation

Inhalation studies											
Duration	CNT					Dose	Experimental model			Fibrosis	References
	Type	Source	Length (μm)	Diameter (nm)	Other		Species	Strain	Methods		
1-28d	SWCNT	CNI	0.1–1	0.8–1.2		5 mg/m^3^, 5 h/d, 4d	mouse	C57BL/6	SSCK, SRS	+	[81]
						whole body					
1y	SWCNT	U	1–3	65		5 mg/m^3^, 5 h/d, 4d	mouse	C57BL/6	SSCK	+	[83]
						whole body					
2-12d	MWCNT-7	HCC	/	/		10 mg/m^3^, 5 h/d	mouse	C57BL/6 J	MTS	+	[84]
						whole body					
1 to 336d	MWCNT-7	HCC	4.3	/		5 mg/m^3^, 5 h/d, 12d	mouse	C57BL/6 J	SRS	+	[27]
						whole body					
17 m	MWCNT-7	HCC	1–6	40–90		5 mg/m^3^, 5 h/d, 5d/w	mouse	B6C3F1	H&E	↑	[85]
						whole body					
90d	MWCNT-7	HCC	5.5–6.2	94–98		0.2–5 mg/m^3^, 5 h/d, 4d	rat	F344	MTS	++	[86]
						whole body					
1d-14w	MWCNT	HMS	0.3–50	30–50		1–30 mg/m^3^, 6 h/d	mouse	C57BL/6	MTS	++	[87]
						nose inh					
90d	MWCNT	N	0.1–10	5–15		0.1–2.5 mg/m3,	rat	Wistar	H&E	-	[88]
						6 h/d, 5d/w, 13w					
						nose inh					
90d	MWCNT	N	0.1–10	5–15		0.1–2.5 mg/m^3^	rat	SH	MTS, LS, GS	-	[89]
						6 h/d, 5d/w, 13w					
						nd inh					

nd: not determined. *Duration:* d: day; w: week; y: year. *CNT type:* AP: as prepared; COOH: carboxyl; NH2: amino; PD: purified. Source: A: Arkema (France); CNI: Carbon Nanotechnologies Inc. (Houston, TX); CT: Cheap Tubes (Brattlebore, VT); HCC: Hodogaya Chemical Company; HMS: Helix Materials Solution (Richardson, TX); MC: Mitsui & Company (Tokio, Japan); N: Nanocyl (Sambreville, Belgium); NI: NanoIntegris (Skokie, IL); NP: Nanotech Port (Chengdu, China); PIH: produced in-house; SA: Sigma-Aldrich (Lyon, France); SN: Shenzhen Nanoharbor (Shenzhen, China); SNP: Shenzhen Nanotech Port (Shenzhen, China); U: Unidym (Sunnyvale, CA). *Other:* AD: acetone/sonication dispersed; ALD: atomic layer deposition; BSA: bovine serum albumin; cc-PEI: carboxyl converted PEI; D: dispersed; DPPC: dipalmitoylphosphatidylcholine; F: functionalized; ND: no dispersed; PABS: polyaminobenzene sulfonic acid group; PEG: polyethylene glycol; PEI: polyethyleneimine; PF108-C: cruder stock; PF108-HD: homogeneously dispersed; SD: survanta dispersed; swNH2: sidewall amine. *Methods:* GS: Gomori staining; H&E: Hematoxylin and eosin stain; hcIll: histopathology collagen type III; LS: Ladewig staining; MTS: Masson's trichrome staining; OH-p: hydroxyl proline, SRS: Sirius red staining; SSCK: Sircol Soluble Collagen; WBc: western blot collagen. Fibrosis: +*p* < 0.05, ++*p* < 0.01, +++*p* < 0.001 as reported in the respective paper; (#) *p* < 0.05 difference between particle responses as reported in the respective paper; ↑: increase of fibrosis suggested by authors but without statistical evidence

**Table 2:** In vivo lung fibrotic reaction of CNT administered via intratracheal injection

Intratracheal injection studies											
Duration	CNT					Dose	Experimental model			Fibrosis	References
	Type	Source	Length (μm)	Diameter (nm)	Other		Species	Strain	Methods		
7-56d	short SWCNT	SN	0.35–0.7	10–20		60 μg	Mouse	C57BL/6 J	SSCK, MTS	-	[65]
	long SWCNT		5–15	10–20		(3 mg/kg)				+	
14d	SWCNT AMIDE	/	0.7–1	4–6	F	10 mg/kg	Mouse	C57BL/6	MTS, hcI, hcIII, hα-SMA	++	[90]
	SWCNT COOH		0.5–1.5	4–5	F					++	
	SWCNT PABS		0.5-1	1.1	F					++	
	SWCNT PEG		0.5–0.6	4.5	F					++	
1-180d	MWCNT	SA	0.5–2	20–50	BSA disp	1–100 μg	Rat	Sprague–Dawley	SSCK, MTS	-	[91]
						(5–500 μg/kg)					
1-91d	MWCNT-7	MC	5	88		40–160 μg	Rat	F344	MTS	↑	[92]
						(0.2–0.8 mg/kg)					
1-30d	short MWCNT	NP	0.5–2	50		0.6 mg	Rat	SH	SRS, hcIII	-	[15]
	long MWCNT		20–50	50		(3 mg/kg)				++	
60d	MWCNT	PIH	5.9	9.7		0.5–5 mg	Rat	Sprague–Dawley	OH-p, MTS, ELISAc	+++	[2]
	MWCNTg		0.7	11.3		(2.5–25 mg/kg)				++	
60d	MWCNTg	PIH	0.7	20–50	defects +++	2 mg	Rat	Wistar	OH-p, MTS	++	[93]
	MWCNTg 600				defects ++	(10 mg/kg)				+++	
	MWCNTg 2400				defects +					+++	

nd: not determined. *Duration:* d: day; w: week; y: year. *CNT type:* AP: as prepared; COOH: carboxyl; NH2: amino; PD: purified. Source: A: Arkema (France); CNI: Carbon Nanotechnologies Inc. (Houston, TX); CT: Cheap Tubes (Brattlebore, VT); HCC: Hodogaya Chemical Company; HMS: Helix Materials Solution (Richardson, TX); MC: Mitsui & Company (Tokio, Japan); N: Nanocyl (Sambreville, Belgium); NI: NanoIntegris (Skokie, IL); NP: Nanotech Port (Chengdu, China); PIH: produced in-house; SA: Sigma-Aldrich (Lyon, France); SN: Shenzhen Nanoharbor (Shenzhen, China); SNP: Shenzhen Nanotech Port (Shenzhen, China); U: Unidym (Sunnyvale, CA). *Other:* AD: acetone/sonication dispersed; ALD: atomic layer deposition; BSA: bovine serum albumin; cc-PEI: carboxyl converted PEI; D: dispersed; DPPC: dipalmitoylphosphatidylcholine; F: functionalized; ND: no dispersed; PABS: polyaminobenzene sulfonic acid group; PEG: polyethylene glycol; PEI: polyethyleneimine; PF108-C: cruder stock; PF108-HD: homogeneously dispersed; SD: survanta dispersed; swNH2: sidewall amine. *Methods:* GS: Gomori staining; H&E: Hematoxylin and eosin stain; hcIll: histopathology collagen type III; LS: Ladewig staining; MTS: Masson's trichrome staining; OH-p: hydroxyl proline, SRS: Sirius red staining; SSCK: Sircol Soluble Collagen; WBc: western blot collagen. Fibrosis: +*p* < 0.05, ++*p* < 0.01, +++*p* < 0.001 as reported in the respective paper; (#) *p* < 0.05 difference between particle responses as reported in the respective paper; ↑: increase of fibrosis suggested by authors but without statistical evidence

**Table 3:** in vivo lung fibrotic reaction of CNT administered via pharyngeal aspiration

Pharyngeal aspiration studies											
Duration	CNT					Dose	Experimental model			Fibrosis	References
	Type	Source	Length (μm)	Diameter (nm)	Other		Species	Strain	Methods		
2w	SD SWCNT	CNI	0.1–1	0.8–1.2	survanta acetone disp	10 μg	mouse	C57BL/6 J	SSCK, WBc	+	[29]
	AD SWCNT					(0.5 mg/kg)				+	
1-28d	SWCNT	CNI	0.1–1	0.8–1.2		5–20 μg	mouse	C57BL/6	SSCK, SRS	↑	[81]
						(0.25–1 mg/kg)					
1-60d	SWCNT	CNI	/	1–4		10–40 μg	mouse	C57BL/6	SRS, MTS	+	[38]
						(0.5–2 mg/kg)					
28d	SWCNT	CNI	/	1–4		40 μg	mouse	C57BL/6	SSCK	+	[17]
						(2 mg/kg)		C57BL/6gp91^phox−/−^		-	
28d	SWCNT	CNI	0.7	1–4		40 μg	mouse	C57BL/6	SRS, SSCK	+	[94]
						(2 mg/kg)		B6.129X1-MPO		+ (#)	
3w	SWCNT	CNI	0.5–2	0.4–1.2		40 μg	mouse	C57BL/6	SSCK, MTS	+	[95]
						twice a week 3 weeks					
7d	SWCNT	CNI	1–3	1–4		40–80 μg	mouse	C57BL/6	SSCK	+	[96]
						(2–4 mg/kg)					
28d	SWCNT	U	1–3	1–4		40 μg	mouse	C57BL/6	SRS, SSCK	+	[19]
						(2 mg/kg)					
1y	SWCNT	U	1–3	65		40 μg	mouse	C57BL/6	SSCK	+	[83]
						(2 mg/kg)					
21d	SWCNT BSA-AP-Hipco	NI	1000–5000	0.7–1.1	BSA-coated	2 mg/kg	mouse	C57BL/6	SSCK, MTS	+	[47]
	SWCNT PF108-Hipco		500–1500	0.7–1.1	PF-108-coated					-	
14-56d	SWCNT	/	5–15	<2		80 μg/m	mouse	C57BL/6	OH-p, MTS	+	[64]
						(4 mg/kg)					
90d	Short SWCNT	CT	1	11		40 μg/m	mouse	C57BL/6 J	SSCK, SRS	+	[31]
	Long SWCNT		13	11		(2 mg/kg)				+ (#)	
21d	MWCNT	CT	10–30	20–30		2 mg/kg	mouse	C57BL/6	SSCK, MTS	+	[54]
								P47^phox−/−^ C57BL/6		-	
21d	MWCNT AP	CT	0.57		F					+	
	MWCNT NH2	From AP	0.45		F					+	
	MWCNT sw-NH2	From AP	0.58		F					+	
	MWCNT PEI	From AP	0.33	/	F	2 mg/kg	mouse	C57BL/6	SSCK, MTS	+ (#)	[45]
	MWCNT cc-PEI	From AP	-		F					+	
	MWCNT COOH	From AP	0.38		F					-	
	MWCNT PEG	From AP	0.27							-	
21d	AP MWCNT D	CT			BSA + DPPC					+	
	AP MWCNT ND	CT			/					-	
	PD MWCNT D	From AP	10–30	20–30	BSA + DPPC	2 mg/kg	mouse	C57BL/6	MTS, SSCK	+	[36]
	PD MWCNT ND	From AP			/					-	
	COOH MWCNT D	From AP			BSA + DPPC					+	
	COOH MWCNT ND	From AP			/					-	
21d	AP MWCNT	CT	1.97		BSA-dispersed					+	
	AP MWCNT	CT	1.16		PF108-C					-	
	AP MWCNT	CT	0.15		PF108-HD					-	
	PD MWCNT	From AP	2.11		BSA-dispersed					+	
	PD MWCNT	From AP	1.52	20–30	PF108-C	2 mg/kg	mouse	C57BL/6	SSCK, MTS	-	[97]
	PD MWCNT	From AP	0.14		PF108-HD					-	
	COOH MWCNT	From AP	1.96		BSA-dispersed					+	
	COOH MWCNT	From AP	1.8		PF108-C					-	
	COOH MWCNT	From AP	0.15		PF108-HD					-	
1-14d	MWCNT-7	MC	3.9	49		5–40 μg	mouse	C57BL/6 J	MTS, SRS	+++	[37]
						(0.25–2 mg/kg)					
1-56d	MWCNT-7	MC	3.86	49		10–80 μg	mouse	C57BL/6 J	SRS	+	[26]
						(0.5–4 mg/kg)					
1-56d	MWCNT-7	MC	3.86	49		10–80 μg/m	mouse	C57BL/6 J	SRS	+	[82]
						(0.5–4 mg/kg)					
1 to 56d	MWCNT-7	HCC	4.3	/		80 μg/m	mouse	C57BL/6 J	SRS	+	[27]
						(4 mg/kg)					
28d	MWCNT	HMS	0.5–40	10–30	Uncoated	4 mg/kg	mouse	C57BL/6	MTS	+++ (#)	[50]
					ALD-coated					+	
60d	MWCNT NM400	N	0.7–3	5–35						+++	
	MWCNT NM402	A	0.7–4	6–20		12.5–100 μg/m	mouse	C57B6/6	OH-p, SRS	+++	[23]
	MWCNT NM400c	N	0.1–0.5	18–35		(0.6–5 mg/kg)				-	
	MWCNTg 2400	PIH	0.7	20–50						-	
60d	MWCNT NM400	N	0.7–3	5–35						++	
	MWCNT long	CT	10–30	11–59		100 μg/m	mouse	C57BL/6	OH-p, SRS	++	[34]
	MWCNT short	CT	0.5–2	10–47		(5 mg/kg)				-	
	MWCNT thick	CT	10–30	15–74						-	

nd: not determined. *Duration:* d: day; w: week; y: year. *CNT type:* AP: as prepared; COOH: carboxyl; NH2: amino; PD: purified. Source: A: Arkema (France); CNI: Carbon Nanotechnologies Inc. (Houston, TX); CT: Cheap Tubes (Brattlebore, VT); HCC: Hodogaya Chemical Company; HMS: Helix Materials Solution (Richardson, TX); MC: Mitsui & Company (Tokio, Japan); N: Nanocyl (Sambreville, Belgium); NI: NanoIntegris (Skokie, IL); NP: Nanotech Port (Chengdu, China); PIH: produced in-house; SA: Sigma-Aldrich (Lyon, France); SN: Shenzhen Nanoharbor (Shenzhen, China); SNP: Shenzhen Nanotech Port (Shenzhen, China); U: Unidym (Sunnyvale, CA). *Other:* AD: acetone/sonication dispersed; ALD: atomic layer deposition; BSA: bovine serum albumin; cc-PEI: carboxyl converted PEI; D: dispersed; DPPC: dipalmitoylphosphatidylcholine; F: functionalized; ND: no dispersed; PABS: polyaminobenzene sulfonic acid group; PEG: polyethylene glycol; PEI: polyethyleneimine; PF108-C: cruder stock; PF108-HD: homogeneously dispersed; SD: survanta dispersed; swNH2: sidewall amine. *Methods:* GS: Gomori staining; H&E: Hematoxylin and eosin stain; hcIll: histopathology collagen type III; LS: Ladewig staining; MTS: Masson's trichrome staining; OH-p: hydroxyl proline, SRS: Sirius red staining; SSCK: Sircol Soluble Collagen; WBc: western blot collagen. Fibrosis: +*p* < 0.05, ++*p* < 0.01, +++*p* < 0.001 as reported in the respective paper; (#) *p* < 0.05 difference between particle responses as reported in the respective paper; ↑: increase of fibrosis suggested by authors but without statistical evidence

Regulatory toxicology currently moves towards the mode of action concept, aligned with so-called adverse outcome pathways (AOP). AOP refers to a conceptual structure that portrays existing knowledge concerning a series of key events (KE) along a biological pathway from the molecular initial event (MIE) to the adverse outcome (AO) [9]. Organization of these data in an AOP provides a systematic and transparent knowledge that may help elucidating and identifying the key mechanisms of toxicity, and the strength of their relationship, which lead to the progression of the AO. This knowledge can guide researchers to focus on key toxic events and assays or, where data gaps exist, this might provide an incentive to identify and highlight new mechanisms of action. Additionally, AOPs can facilitate the development of simple and fast test methods to predict the potential hazard of CNT, thus reducing the use and costs of animal and time-consuming experiments. The present review focuses on mechanisms of the lung fibrotic reaction induced by CNT with a view to developing an AOP.

## Lung fibrotic reactions and CNT

In response to prolonged tissue injury or chronic inflammation, fibroblasts can be activated, resulting in three KE involved in the development of lung fibrosis: i) cell proliferation, ii) differentiation into myofibroblasts and iii) exaggerated production of extracellular matrix (ECM) proteins, leading to ECM accumulation in the lung parenchyma, modification of the lung architecture, organ dysfunction and, eventually, severe respiratory insufficiency [10]. Fibroblasts can derive from different sources, including lung resident fibroblasts, epithelial cells undergoing epithelial-mesenchymal transition (EMT) and bone marrow progenitors called fibrocytes. Numerous studies have aimed at identifying how CNT can promote pro-fibrotic responses, based on the current paradigm in particle toxicology that considers particle-inflammation as key player in inducing fibrosis [11, 12].

CNT can induce the release of pro-inflammatory and pro-fibrotic growth factors by macrophages and epithelial cells [13–16], which are in the frontline in lung alveoli. The release of these mediators can induce the recruitment, proliferation and differentiation of fibroblasts, which then produce the ECM components. In this context, interstitial fibroblasts, epithelial cells undergoing EMT and fibrocytes can be involved. Additionally, other cell types seem to be involved in fibrogenic responses induced by CNT, such as T and B cells, polymorphonuclear neutrophils (PMN), mast and endothelial cells [17–21]. However, there is no evidence on how these cells can activate fibroblasts upon CNT exposure and lead to fibrosis.

In addition, scientists recently started to scrutinize the potential interaction of CNT with fibroblasts. CNT can activate fibroblasts in vitro, resulting in increased proliferation, differentiation and collagen production [22–24]. Interestingly, only fiber-shape particles such as asbestos [25] and CNT seem to have the capacity to induce fibroblast activation, suggesting a new mechanism of action for these particles.

After reviewing recent advances in this area we propose an AOP, focusing on CNT and lung fibrosis. In agreement with the observations reported above, we classified the effects of CNT in two different ways: i) direct effects on fibroblasts and ii) pro-inflammatory and pro-fibrotic effects on macrophages and epithelial cells, which we call “indirect” effects on fibroblasts. For the indirect effect on fibroblasts, we focused on macrophages and epithelial cells, because of their central role and extensive literature published in this area. To have a clear overview of the pathways involved in the development of fibrosis we further structured the review in multiple sections for each cell type analyzed (CNT localization, effects, mechanisms, relevance of in vitro CNT activities for in vivo effects) in order to design a structured AOP. We summarize here evidences of a close cooperation between fibroblasts, macrophages and epithelial cells in the development of pulmonary fibrosis upon CNT exposure.

## The direct effects of CNT on fibroblasts

### Fibroblasts and CNT lung localization

To support the new paradigm that CNT can have a direct effect on fibroblasts, we first need to address the localization of CNT deposited in the lung. Recent studies have shown the localization of CNT in the alveolar interstitium after pharyngeal aspiration or inhalation exposure using light microscopy, field emission electron microscopy (FESEM) and a new technique based on enhanced-darkfield illumination optics and imaging [26, 27]. These studies support the idea that CNT are able to reach the alveolar interstitium rapidly after exposure and get in close contact with fibroblasts, suggesting that these particles can act directly on these cells possibly contributing to fibrosis development. This observation is further supported by a previous paper by Mercer et al. [28], who observed that dispersed SWCNT (average equivalent diameter 0.69 μm) reached the deep lung and rapidly penetrated in the alveolar interstitium causing after 7 days an increase of thickness of alveolar walls and collagen content after pharyngeal aspiration of 10 μg CNT/mouse (corresponding to 0.5 mg/kg b.w., considering a mouse weight of 20 g). On the contrary, agglomerates and poorly dispersed SWCNT (average equivalent diameter 15.2 μm) were principally deposited in the proximal alveolar region and formed granuloma, confirming that CNT aggregation plays a role in their tissue distribution and thus fibrogenic activity. These studies used doses of CNT equivalent to a human exposure scenario for a person performing a light work, based on average daily CNT workplace exposure. Also the group of Wang et al. [24] showed that MWCNT can directly interact with fibroblasts in vivo. Long MWCNT (length 20–50 μm), which were not phagocytosed by macrophages, were found at the surface and also inside cells stained positively for FSP-1 (fibroblast specific marker) contrary to short MWCNT (length 0.5-2 μm) after intratracheal instillation of 0.6 mg/rat (3 mg/kg b.w., considering a rat weight of 200 g). This dose is similar to that used by Mercer et al. [28] as 80 μg CNT/mouse corresponds to 4 mg/kg b.w. Taken together, these data confirm the relevance of investigating the direct activities of CNT on lung fibroblasts.

### CNT activities on fibroblasts

#### Effects

**Fig. 1:**
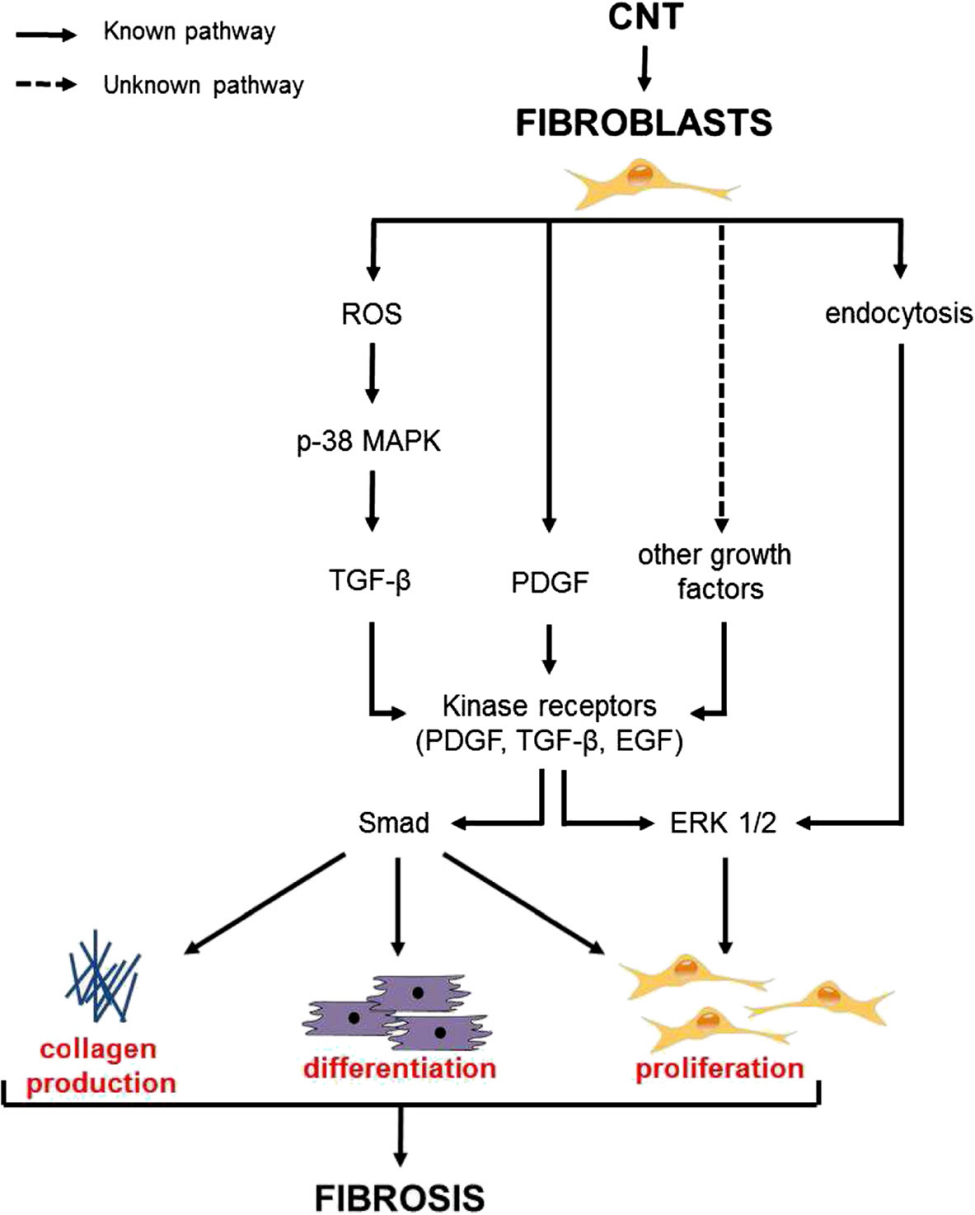
Direct effects of CNT on fibroblasts

In this section, we focus on in vitro studies that addressed the activation of fibroblasts by CNT, as illustrated in Fig. 1. As mentioned above, the ultimate events in fibrogenesis are fibroblast proliferation, differentiation and ECM production. The first study that demonstrated a direct effect of CNT on fibroblasts was published by the group of Wang et al. [22]. Low concentrations of dispersed SWCNT (0.02–0.24 μg/cm^2^), extrapolated from in vivo doses (10–40 μg/mouse, corresponding to 0.5–2 mg/kg b.w.), stimulated cell proliferation, collagen production and matrix metalloproteinases (MMP)-9 expression in human lung fibroblast (CRL-1490). Other in vitro studies observed that both SWCNT and MWCNT were able to directly stimulate fibroblast proliferation, differentiation and collagen production [14, 23, 24, 29–31]. In general, these studies demonstrated that CNT have a direct and rapid effect on different types of fibroblasts, only after 24 or 48 h exposure to doses of CNT relevant for in vivo and human exposure scenarios. These results also show that CNT activity on fibroblasts is not species-specific and that the types of fibroblast tested, such as CRL-1490 and mouse lung fibroblast (MLg), are useful cell models, providing support to extrapolate in vitro data to in vivo and human exposures. However, some CNT have also been observed to be cytotoxic to fibroblasts [13, 32] and it appears that this effect is related to the production of reactive oxygen species (ROS) by fibroblasts exposed to CNT, which we will describe in more details in the next section. MWCNT (20 μg/ml) were also found to increase transforming growth factor (TGF)-β and platelet-derived growth factor (PDGF) mRNA and proteins in lung fibroblasts after 24 h exposure [13]. Both growth factors are well known as key mediators in the pathogenesis of pulmonary fibrosis. PDGF stimulates fibroblast proliferation and acts as a potent chemoattractant for fibroblasts and myofibroblasts, thereby promoting fibrosis. TGF-β stimulates the differentiation of mesenchymal cells and the synthesis of collagen. Also, pro-inflammatory mediators could mediate the release of TGF-β and PDGF and thus potentially exacerbate fibrosis development [13]. However, the release of pro-inflammatory mediators by fibroblasts after CNT exposure has not been observed so far.

#### Mechanisms

Few studies investigated the specific mechanisms of action by which CNT can directly promote fibroblast activation. A recent study observed that long MWCNT (length 20–50 μm, doses 15–60 μg/ml) directly promote fibroblast differentiation into myofibroblasts through the activation of TGF-β/Smad signaling [24]. This pathway is largely known to drive myofibroblast differentiation, but explored for the first time in the context of CNT-induced fibroblast activation. The mechanisms involved in fibroblast proliferation were investigated in two other studies. Azad et al. [14] observed that SWCNT (5–25 μg/ml) induce fibroblast proliferation and collagen production through a ROS-dependent p38 MAPK signaling leading to the production of TGF-β and vascular endothelial growth factor (VEGF). Oxidative stress reflects an imbalance between the production of different reactive metabolites, including ROS and antioxidant defenses. CNT have been shown to induce ROS production both in vitro and in vivo, directly via physico-chemical reactivity, for example through active residual metal catalysts present on the surface of CNT (discussed in section: “Indirect effects of CNT on fibroblasts”), or indirectly, via the activation of enzymatic pathways leading to release of oxidative species. Oxidative stress has been identified as an initiating factor in the pathogenesis of fibrosis as it can increase pro-fibrotic growth factors production leading to the activation of fibroblasts, as described by Azad et al. [14]. Interestingly, Manke et al. [31] also observed that SWCNT (0.06 μg/cm^2^) induced collagen and TGF-β production via ROS generation in normal human lung fibroblasts (NHLF) and these effects were inhibited in the presence of N-acetyl cysteine (NAC). MWCNT-induced oxidative response was also related to a cytotoxic activity of CNT on fibroblasts [32, 33]. We showed the implication of TGF-β, PDGF and epidermal growth factor (EGF) receptors in MWCNT-induced fibroblast proliferation. In presence of low concentrations of different growth factors, we observed that long and thin MWCNT (MWCNT length 10–30 μm and diameter 10–60 nm; doses 15 μg/cm^2^, corresponding to 24 μg/ml by considering a well surface area of 0.32 cm^2^), but not short or thick CNT (MWCNT short: length 0.5-2 μm and diameter 10–50 nm; MWCNT thick: length 10–30 μm and diameter 15–75 nm), directly stimulated fibroblast proliferation via kinase receptors, ERK 1/2 signaling and endocytosis pathways [34]. Some other studies have shown that CNT can be taken up by fibroblasts [24]. However, for the first time it was shown that endocytosis is required for the activation of fibroblasts [34]. Moreover, kinase receptors have been found to be involved in the proliferative activity of MWCNT by using receptor inhibitors, which also inhibited ERK 1/2 signaling pathway [34]. Currently, no data is available on how CNT could interact with these receptors. Asbestos fibers have been shown to directly bind and activate EGF receptor [35], suggesting that CNT could somehow interact with kinase receptors. Azad et al. [14] and Vietti et al. [34] have identified different mechanisms leading to CNT-induced fibroblast proliferation. This may be related to the physico-chemical properties of CNT, suggesting that CNT can activate fibroblast in different ways depending on their structure and possibly their potential to generate ROS. However, these differences can also reflect different experimental procedures, such as cell lines, dispersion medium and methods used. It should be noted that the doses able to activate fibroblasts are in the same range of 5–60 μg/ml, which has been calculated to be relevant for in vivo and human exposure scenarios.

#### Relevance of in vitro CNT activities for in vivo effects

Exploring correlations between in vitro results and pulmonary fibrotic responses in vivo is helpful to derive in vitro assays suitable for predicting the lung fibrogenic activity of CNT. Several studies observed a relationship between in vitro results on fibroblasts and in vivo findings [23, 30, 31, 36]. The capacity of a range of CNT to induce fibroblast proliferation and collagen production has been shown to correlate with their in vivo fibrotic activity. Moreover, the upregulation of TGF-β expression by fibroblasts in vitro upon CNT exposure has been associated with the in vivo lung fibrosis outcome [31]. More recently, MWCNT-induced prolongation of ERK 1/2 signaling pathway in vitro was reported as a mechanism correlated with their activity on lung fibroblast proliferation and, most interestingly, with their lung fibrotic activity in vivo [34]. Taken together, these results show that in vitro responses of lung fibroblasts to CNT correlate to their pro-fibrogenic potential in vivo. Assessing the activity of CNT in fibroblast proliferation and collagen production assays may therefore contribute to refine, reduce and/or replace conventional in vivo animal tests for predicting pulmonary fibrotic responses.

## Indirect effects of CNT on fibroblasts

The fibrogenic activity of inhaled particles, such as asbestos and crystalline silica, is usually ascribed to an indirect effect on fibroblasts mediated by macrophages and epithelial cells. In this section, we focus on the role of these cells in promoting, indirectly, fibroblast activation upon CNT exposure.

### The role of macrophages

Macrophages are inflammatory cells involved in innate and adaptive immunity, present in alveoli as front line immune defense. Their primary role is to remove or sequester offending agents. They play an essential role in intercellular communication through the release of growth factors and cytokines promoting lung fibrosis including the pro-fibrotic growth factors PDGF and TGF-β, and pro-inflammatory cytokines, such as interleukin (IL) -1β.

#### Macrophage recruitment and CNT phagocytosis

Traditionally, particle-induced fibrosis (asbestosis and silicosis) has been associated with sustained inflammatory recruitment and responses as a first step in fibrogenesis. Dong et al. [37] have shown a rapid infiltration of macrophages in lung tissues and alveoli after pharyngeal aspiration of CNT (40 μg/mouse, corresponding to 2 mg/kg b.w.). They observed a macrophage infiltration at day 1 post-exposure that was maintained till day 7 and then reduced but significantly higher than in controls through day 14 in both the interstitial and alveolar spaces. The early macrophage infiltration was also confirmed in other studies with an up-regulated number until day 28, accompanied by an early increase of pro-inflammatory cytokines [18, 38]. Mangum et al. [39] did not observe an inflammatory response upon SWCNT exposure (2 mg/kg rat b.w.) after 21 days, although interstitial fibrotic lesions were observed near clusters of macrophages that had engulfed aggregates of SWCNT. However, it should be noted that lung histology may not be sufficiently sensitive to exclude SWCNT-induced inflammatory responses. Interestingly, the authors observed that CNT were able to form intercellular structures that bridged alveolar macrophages at 21 days post-exposure, while these structures were not observed in the lungs of rats exposed to carbon black (CB). Rats were exposed to equivalent doses of SWCNT and CB (2 mg/kg b.w.), with similar specific surface area (about 300–600 m^2^/g), thus suggesting that the intercellular structures that bridged alveolar macrophages were probably due to particle shape. How CNT bridges form and their functional consequences have yet to be determined. They observed that a major portion of CNT was engulfed by macrophages. Others papers have also observed the uptake and long-term persistence of CNT within macrophages [2, 40]. Mercer et al. [27, 28, 40] documented the presence of CNT within alveolar and interstitial macrophages and the pulmonary persistence of these particle was associated with toxicity. However, in the study of Kobayashi et al. [41], fibrotic responses were not observed, though MWCNT (0.04-1 mg/kg rat b.w.) were present in alveolar macrophages until 6 month post-exposure. Authors suggested that MWCNT were being processed and cleared by alveolar macrophages probably due to their short length (1.5 μm). It could also be due to the lower doses used compared to other studies (Table 1, 2, 3).

Collectively, these observations show that CNT interact with macrophages. We discuss below the activities of CNT on macrophages and their mechanisms of action and how this may impact on fibroblast activation, thus contributing to the indirect pro-fibrotic effects of CNT on fibroblasts as part of the AOP.

#### CNT activities on macrophages

##### Effects

Macrophages can, in response to CNT, produce pro-inflammatory and pro-fibrotic mediators that play important roles in inflammation and establishment of pulmonary fibrosis. Among pro-inflammatory mediators, IL-1β has been reported to play a key role in fibrosis pathogenesis. MWCNT (25–100 μg/ml) were shown to induce the release of IL-1β and IL-18 by cultured alveolar macrophages isolated from naive C57BL/6 mice and by human monocytic cell line (THP-1), and their secretion was suppressed by inhibitors of caspase-1 and cathepsin B [42]. Several other in vitro studies observed that CNT enhanced IL-1β release in macrophages via inflammasome activation, nuclear factor-kappa B (NF-kB) and oxidative stress [13, 35, 36, 43–47], which we discuss in the mechanisms section. Moreover, IL-1β has also been shown to induce the release of the pro-fibrotic mediators TGF-β and PDGF from different cell types, thus highlighting the importance of the pro-inflammatory mediators released by macrophages [13]. Tumor necrosis factor (TNF)-α is also known to upregulate the expression of TGF-β by epithelial cells [48]. Increased levels of TNF-α were observed in murine lung after CNT administration and secreted by macrophages after in vitro exposure to CNT [2, 38, 49, 50]. However, the role of TNF-α in CNT-induced fibrosis is less well documented than for IL-1 β.

Both SWCNT and MWCNT have also been shown to induce the secretion of TGF-β and PDGF from macrophages in vitro and in vivo [13, 15, 38, 45, 51]. Conditioned media from macrophages exposed to CNT, in which TGF-β and PDGF was measured, acted as paracrine signals on fibroblasts by inducing their activation, thus potentially contributing to the development of CNT-induced lung fibrosis [13, 15]. Recent reports also demonstrated the release of osteopontin (OPN), MMP and tissue inhibitors of matrix metalloproteinase (TIMP) by macrophages [50, 52]. The pro-fibrotic activity of OPN is known to involve proliferation and migration of fibroblasts and, like MMP and TIMP, modulation of ECM. However, evidence for the role of OPN in CNT-induced fibroblast activation is currently lacking.

Thus, we identified IL-1β, TGF-β and PDGF released by macrophages as relevant paracrine signals to stimulate fibroblasts via alteration of several signal transduction pathways that may contribute to the AOP.

##### Mechanisms

Recent studies have reported the role of inflammasome activation in inducing pulmonary inflammation and fibrosis. Inflammasome is a multiprotein oligomer that mediates activation of caspase-1 which promotes the maturation of the pro-inflammatory cytokines IL-1β and IL-18. Members of the Nod-like receptor family, including NLRP1, NLRP3 or NLRC4, and the adaptor ASC are critical components of the macrophage inflammasome. Several studies observed that CNT enhance IL-1β release by macrophages via inflammasome activation [42–46].

Nuclear factor-kappa B (NF-kB), a key mediator of inflammatory responses, is another player implicated in the release of IL-1β. He et al. [13] showed that MWCNT (2–20 μg/ml) increased the secretion of different pro-inflammatory cytokines and chemokines, such as IL-1β, TNF-α, IL-6, IL-10 and MCP1, through the activation of the NF-κB signaling pathway in mouse leukaemic monocyte macrophage cell line (RAW 264.7). However, authors did not provide data linking the release of pro-inflammatory mediators with the activation of NF-kB. Thus, it remains critical to elucidate the mechanisms by which CNT affect NF-kB activity and the downstream responses, as the release of IL-1β. Similar observations were obtained after THP-1 exposure to SWCNT (0.05 mg/ml), which led to the activation of NF-kB and the secretion of different cytokines [53].

Activation of the inflammasome and release of IL-1β have also been associated with oxidative stress induced by CNT. Nicotinamide adenine dinucleotide phosphate (NADPH) oxidase is an important regulator of inflammasome and fibrotic development in response to CNT. NADPH oxidase is a membrane-bound enzyme complex that generates superoxide by transferring electrons from NADPH and coupling these to molecular oxygen to produce superoxide anion. NADPH oxidase is composed of transmembrane (gp91^phox^ and p22^phox^), cytosolic (p40^phox^, p47^phox^ and p67^phox^) and GTPase (Rac 1/2) subunits. Sun et al. [54] observed that NADPH oxidase-induced oxidative stress was involved in the generation of lung fibrosis upon MWCNT exposure, via NLRP3 inflammasome activation and IL-1β production. These responses were obtained in wild-type and p22^phox^-deficient THP-1 macrophage cells, p47^phox^-deficient primary bone marrow-derived macrophages and in vivo models (C57BL/6 and p47^phox−/−^ C57BL/6 mice) exposed to 50 μg/ml and 50 μg/mouse (corresponding to 2.5 mg/kg b.w.) MWCNT, respectively. These data were also confirmed by treating wild type mice with NAC, a thiol antioxidant, confirming a significant reduction of IL-1β, TGF-β and collagen deposition in vivo. Another study showed that long, needle-like CNT (100 μg/ml, length of about 13 μm) induced NLRP3 inflammasome activation via ROS production [43]. Thus, oxidative stress plays a role in the activation of the inflammasome by CNT and release of IL-1β, which can contribute to the induction of pro-fibrotic growth factors and fibrosis development.

Phagocytosis of CNT has also been implicated in the release of reactive species, mainly associated with frustrated phagocytosis, which is a pro-inflammatory condition [55]. This occurs when particles are physically too long for the macrophages to engulf them. CNT which undergo frustrated phagocytosis or were taken up by macrophages induced oxidative stress [56, 57]. Further studies should be conducted to investigate the direct relationship between CNT phagocytosis, oxidative stress and their impact on the release of IL-1β, for example by using endocytosis inhibitors. Moreover, frustrated phagocytosis also led to CNT biopersistence, as the clearance of inhaled material is hampered, and to the release of pro-inflammatory (IL-6 and IL-8) and pro-fibrotic mediators (TGF-β) [58, 59]. These data suggest a relevant role of oxidative stress in CNT-induced IL-1β release and fibrosis development.

In conclusion and in the context of the AOP, inflammasome, NF-kB and oxidative stress induced by CNT in macrophages can be identified as important mechanisms of action since these can induce the release of pro-inflammatory and pro-fibrotic mediators regulating fibroblast activation and fibrotic responses.

##### Relevance of in vitro CNT activities for in vivo effects

We found fewer studies that investigated the relationship between the response of macrophages in vitro and in vivo data regarding the development of lung fibrosis, compared to the direct effect of CNT on fibroblasts. CNT able to trigger the release of IL-1β by THP-1 or alveolar macrophages in vitro were observed to induce an increase of this cytokine and collagen deposition in the alveolar region of mice [36, 45, 47, 54]. Sun et al. [54] observed the implication of NADPH oxidase in the activation of the NLRP3 inflammasome and the release of IL-1β both in vitro and in vivo and thus the development of lung fibrosis. Unfortunately, these authors have investigated only one type of MWCNT in vivo whereas several CNT were used in the in vitro tests. Thus, the relevance of these in vitro data should be confirmed with a wider range of CNT. The release of TGF-β and PDGF has also been observed in a co-culture of THP-1 with human bronchial epithelial cells (BEAS-2B) exposed to CNT and associated with an increase of collagen and of these growth factors in vivo [45].

**Fig. 2:**
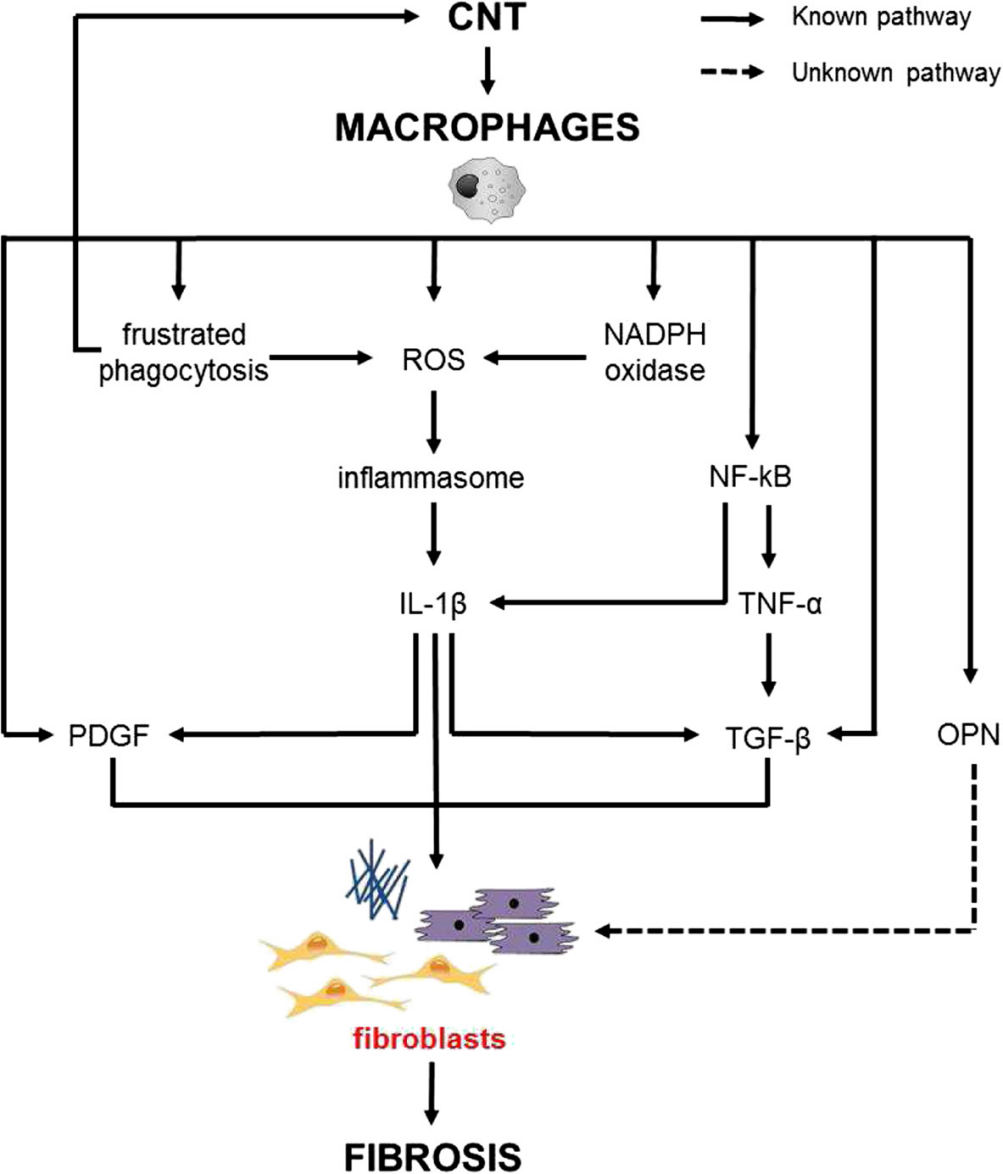
Indirect effects of CNT on fibroblasts via macrophages

Taken together, these results suggest that the release of TGF-β, PDGF and IL-1β (that can also act as autocrine signals) by macrophages exposed to CNT can act as paracrine signals for the activation of fibroblasts, supporting an indirect mechanism of CNT to activate fibroblasts, as illustrated in Fig. 2. This knowledge is used for establishing the AOP, as in vitro studies on macrophages were identified as another potentially effective model for assessing the pro-fibrogenic potential of CNT. To investigate the indirect effect of CNT on fibroblast via macrophages, TGF-β, PDGF and IL-1β are relevant mediators and inflammasome, NF-kB and oxidative stress are relevant pathways.

### The role of epithelial cells

The alveolar epithelium comprises two main cell types: type I and type II pneumocytes. Type I cell represents the gas exchange surface in the alveolus and cover about 97 % of the total alveolar surface area, while type II cells mainly release pulmonary surfactant to optimize conditions for gas exchange. In case of tissue injury, type I cells can be damaged, causing a loss of epithelial barrier and type II cells can i) be activated to release inflammatory mediators, ii) proliferate or differentiate in type I cells to restore the injured basement membranes, iii) undergo epithelial-mesenchymal transition (EMT) as an alternative source of fibroblasts to repair injured tissue. Also the epithelial cells in the trachea or bronchi region have a role in the pulmonary system. These cells secrete mucus, which helps to maintain epithelial moisture and traps particulate material and pathogens moving through the airway. Several evidences show that the effect of CNT on epithelial cells can play an important role in fibrosis development. CNT can induce the indirect activation of fibroblasts via the release of pro-inflammatory and pro-fibrotic mediators by epithelial cells and, additionally, CNT have been found to induce the transformation of epithelial cells through EMT.

#### Epithelial surface and CNT localisation

Alveolar epithelial cells are one of the first targets of CNT once inhaled, and CNT were observed to directly interact with alveolar epithelial cells in vivo few days after particle exposure. CNT (0.6 mg/rat, corresponding to 3 mg/kg b.w.) accumulated and were taken up by epithelial cells stained positively for E-cadherin in vivo, and these data were confirmed in vitro in a rat alveolar type II cell line [24]. Mercer et al. [40] observed a rapid and direct penetration of MWCNT (80 μg/mouse, corresponding to 4 mg/kg b.w.) in type I epithelial cells only one day after mice exposure. In contrast, they rarely observed the penetration of MWCNT in type II epithelial cells. However, several in vitro studies showed the capacity of different type II epithelial cell lines to take up CNT [36, 60–62].

The above data highlight that CNT are able to rapidly enter in contact with epithelial cells, thus potentially inducing a variety of adverse effects on these cells, which can contribute to promote a fibrotic process. In the following sections we will discuss CNT activities on epithelial cells, including the process of EMT, and release of pro-inflammatory and pro-fibrotic mediators, and their impact on fibroblasts. This knowledge contributes to the development of the AOP.

#### CNT activities on epithelial-mesenchymal transition

Epithelial injury can lead to EMT, a process characterized by the transition of epithelial cells to mesenchymal features, by which epithelial cells lose their cell polarity and cell-cell adhesion, and gain migratory and invasive properties. EMT has been suggested to play an important role in fibrosis development, providing an alternative source of fibroblasts and causing a loss of epithelial barrier [63].

Currently, few studies have analyzed the effect of CNT on EMT and its effective role in fibrosis induced by CNT is still unclear. Chang et al. [64] showed that in vivo SWCNT exposure (80 μg/mouse, corresponding to 4 mg/kg b.w.) induced EMT of lung epithelial cells, identified by surfactant protein C (SPC) and N-cadherin (N-cad) markers. Moreover, these epithelial derived fibroblasts have been shown to produce collagen. Authors suggested that this process was induced by the activation of TGF-β/p-Smad2 and β-catenin in epithelial cells. Two other studies confirmed the importance of TGF-β/Smad2 signaling in CNT-induced EMT [24, 65]. Chen et al. exposed mice (60 μg/mouse, corresponding to 3 mg/kg b.w.) and A549 cells (1.4–16 μg/cm^2^, corresponding to 2–26 μg/ml considering a well surface area of 0.32 cm^2^) to long (length 5–15 μm) and short (length 350–750 nm) MWCNT. Wang et al. tested long (length 20–50 μm) and short (0.5–2 μm) MWCNT in rats (0.6 mg/rat, corresponding to 3 mg/kg b.w.) and the rat alveolar type II cell line (RLE-6TN; 30 μg/ml). Both in vivo studies showed that only long MWCNT, but not short fibers, directly interacted with epithelial cells, were taken up by endocytosis and promoted EMT. In vitro data confirmed that only long MWCNT induced EMT through the activation of TGF-β/Smad2 signaling in epithelial cells and CNT were shown to downregulate E-cadherin and upregulate α-SMA, cell-cell adhesion and myofibroblast markers, respectively.

In summary, CNT-induced EMT was confirmed in different species and in epithelial cells of various origins. The mechanism of CNT-induced EMT is similar to that identified for CNT-induced fibroblast differentiation, TGF-β/Smad2 signaling playing a key role in cell transformation in response to CNT. On the other hand, asbestos was found to induce EMT in A549 cells via the MAPK/ERK pathway, not associated with TGF-β signaling activity [66]. Thus, CNT have been shown to increase fibroblast population via the induction of EMT.

#### CNT activities on epithelial cells

##### Effects

Activation of epithelial cells after CNT exposure can result in the release of pro-inflammatory and pro-fibrotic mediators. Hussain et al. [16] demonstrated that the internalization of MWCNT (1.5–24 μg/ml) in primary human bronchial epithelial cells (HBE) induced the release of IL-1β and IL-18, which have been identified as key modulators of pro-fibrotic responses in fibroblasts, including the expression of tenascin (TN)-C (an extracellular matrix glycoprotein), TIMP-1 and OPN. Authors also observed a release of IL-8 from epithelial cells upon CNT exposure, which contributed to the expression of TN-C by fibroblasts. Sweeney et al. [67] observed a release of IL-8, and IL-6, upon exposure to MWCNT (1–50 μg/ml) in a human alveolar type-I like epithelial cell line and primary human alveolar type-II epithelial cells, showing that both type I and II cells can play an active role in pulmonary responses to CNT.

Several studies showed that both SWCNT (100 μg/ml) and MWCNT (10–100 μg/ml), were able to trigger the release of TGF-β from BEAS-2B cells [36, 47] that is able to stimulate fibroblasts and induce EMT. In addition, MWCNT (20 μg/ml) were found to increase the expression and protein levels of the pro-fibrogenic growth factor PDGF in BEAS-2B, as well as TGF-β [13].

Another study also analyzed the impact of CNT on ciliated tracheobronchial epithelium, as these cells play a role in the clearance of inhaled particles by ciliary transport [68]. These authors showed that MWCNT with a length of 3 and 8 μm damaged the ciliated epithelium in the trachea of rats (250 μg/ml in rat, corresponding to 75 μg/rat considering that rats were treated with 0.3 ml) and mouse tracheal epithelial cells in vitro (10 μg/ml) and suggested that these lesions can prevent the clearance of inhaled CNT in the lung and thus lead to a chronic inflammatory process. Moreover, long MWCNT and SWCNT (100 μg/ml; length of 5–9 μm and 0.5–100 μm, respectively) have been shown to cause an impairment of airway epithelium barrier, as demonstrated by a decrease of the trans-epithelial electrical resistance (TEER) on human epithelial Calu-3 cells, contrary to short CNT (length 0.5–2 μm) and CB [69]. As reported by Snyder et al. [70], this reduction in barrier functions can be related to the interference of MWCNT with the cytoskeleton on lung epithelium. Further studies should investigate the effects of CNT on epithelial barrier and ciliated epithelium as this could have a role in CNT biopersistence and lung damage.

In summary, CNT stimulate the secretion of relevant pro-fibrotic and pro-inflammatory mediators, including TGF-β, PDGF, IL-1β and IL-8, by epithelial cells that can indirectly promote the activation of fibroblasts and induce EMT, two relevant sources of pulmonary fibroblasts. Moreover, the loss of epithelial barrier and ciliated epithelium can contribute to CNT biopersistence, resulting in chronic damage and permanence of CNT in the lung. It necessary to note that the majority of these studies, regarding the effect of CNT on epithelial cells, have been performed on bronchial cell type, while few authors have focused on alveolar epithelial cells, which, however, represent one of the first target in CNT exposure.

##### Mechanisms

Few studies investigated the mechanism involved in the release of pro-inflammatory and pro-fibrotic mediators by epithelial cells. Hussain et al. have demonstrated that MWCNT (1.5–24 μg/ml) activated the NLRP3 inflammasome in HBE cells and induced the release of IL-1β and IL-18. Conditioned medium from MWCNT-treated HBE added on human lung fibroblasts increased the expression of OPN, TIMP-1, pro-collagen 1 and TN-C, which were decreased in the presence of IL-1β and IL-18 antibodies and siRNA against NLRP3 inflammasome. These results point out the importance of inflammasome activation and derived mediators in epithelial cells to induce fibroblast activation.

The release of IL-6 and IL-8 has also been investigated and has been found to be associated to JNK, p38 and ERK 1/2 MAPK signaling pathways in transformed human alveolar type-I-like epithelial cells (TT1) and primary human alveolar type-II epithelial cells (ATII) cells upon MWCNT (50 μg/ml) exposure [67]. This result was further confirmed by Hirano et al. [71], who observed NF-κB activation, enhanced MAP kinase phosphorylation and increased IL-6 and IL-8 release in BEAS-2B cells after MWCNT (2–10 μg/ml) exposure.

Signs of oxidative stress have also been observed in epithelial cells and several studies reported free radical formation and antioxidant depletion in this cell system after CNT exposure [60, 61, 72–74]. Interestingly, Nymark et al. [75], observed the formation of a unique and yet unidentified radical in both absence and presence of BEAS-2B by two types of long MWCNT (average of lengths 4 and 6 μm). However, contrary to macrophages, there is no evidence of a correlation between oxidative stress and the release of pro-inflammatory mediators. Additional analysis showed that early intracellular uptake of MWCNT (length 1–5 μm; 24 μg/cm^2^ corresponding to 38 μg/ml, considering a well surface area of 0.32 cm^2^) in BEAS-2B correlated with the generation of ROS measured by DCF-DA [37]. Authors suggested that this can lead to different variations in the cellular homeostasis, including cell proliferation, differentiation and motility. SWCNT and MWCNT induced epithelial cell proliferation [29, 34]. However it is still not possible to determine whether this effect is related to CNT-induced ROS and more studies have to be carried out to understand the mechanisms. Moreover, Hussain et al. [16] showed that CNT-induced ROS were also associated to cytotoxic effects as these authors observed a reduction of human bronchial epithelial death after antioxidant treatment.

Regarding the indirect effect of CNT on fibroblasts via epithelial cells, inflammasome, NF-κB and oxidative stress appear as mechanisms altering signal transduction pathways, as previously indicated for macrophages, by inducing the release of different mediators able to activate fibroblasts.

##### Relevance of in vitro CNT activities for in vivo effects

As for macrophages, few studies investigated the correlation between in vitro and in vivo results regarding the development of fibrosis via epithelial cells. Two studies observed a good correlation between the release of the pro-fibrogenic TGF-β from BEAS-2B exposed to CNT in vitro and the induction of a fibrotic response in vivo [36, 47]. Moreover, CNT-induced EMT in vitro correlated with in vivo studies, suggesting that some fibroblasts involved in the lung fibrosis originate from epithelial cells. Further investigation is, however, required to understand the critical role of epithelial cells in fibrosis.

**Fig. 3:**
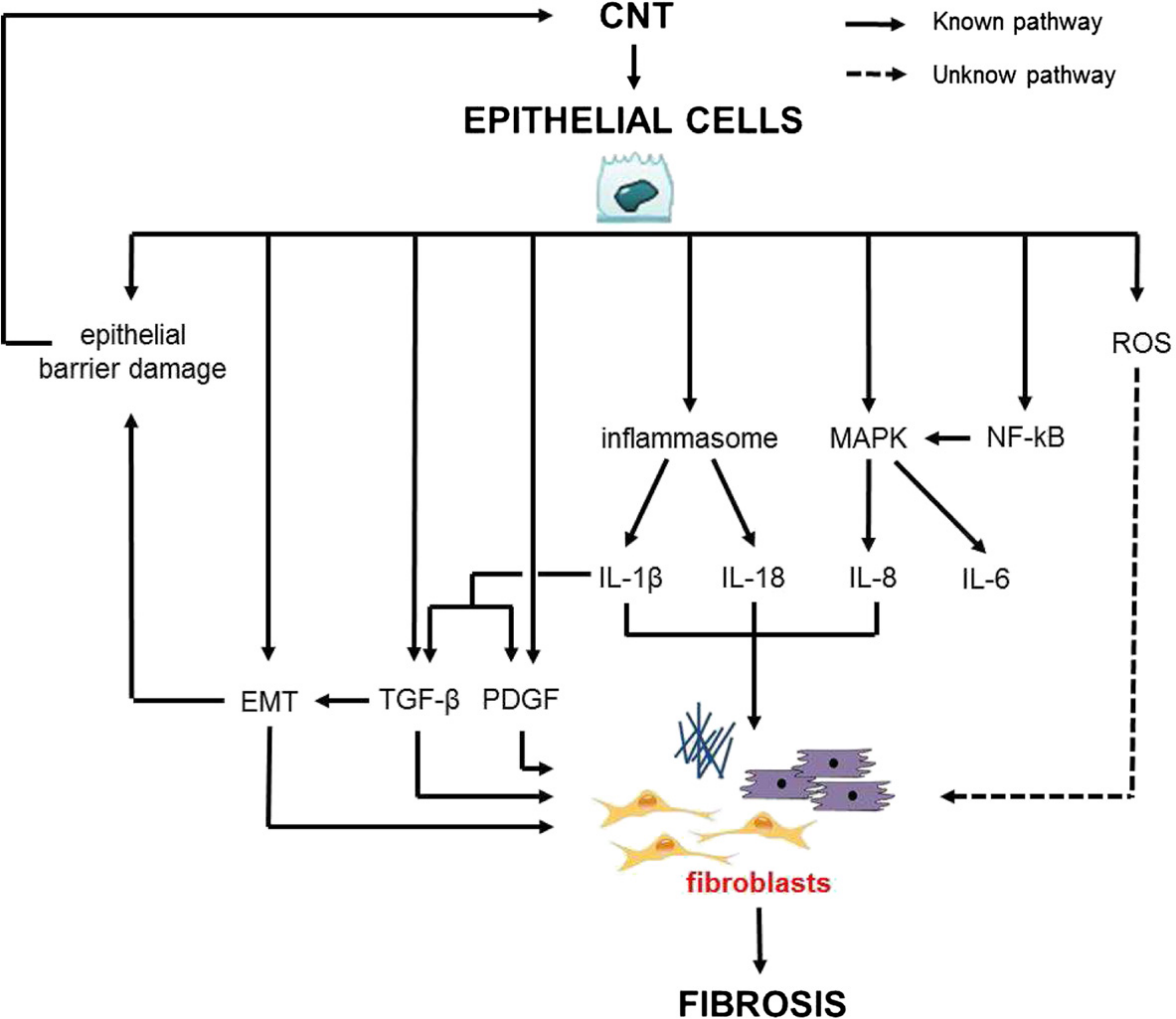
Indirect effects of CNT on fibroblasts via epithelial cells

For the AOP we identify the release of TGF-β from epithelial cells as a key mediator of the indirect effect of CNT on fibroblasts via epithelial cells. However, other relevant mediators, PDGF, IL-1β and IL-8, and pathways, such as inflammasome, NF-kB and oxidative stress, have been shown in vitro to play a role in the complex fibrotic process. Collectively, these data are summarized in Fig. 3.

## Determinants of the fibrotic activities of CNT

Physico-chemical properties of CNT appear to determine their capacity to induce fibroblast, macrophages and epithelial cell activation. The length has been identified as an important factor. Manke et al. [31] showed that long SWCNT (13 μm) were more potent than short SWCNT (1 μm) to elicit a ROS response, collagen production and TGF-β release by fibroblasts in vitro as well as to induce fibrosis in vivo. Several studies confirmed that the length of CNT constitutes an important determinant in the capacity of these materials to induce the activation of fibroblasts and lung fibrosis [24, 34]. Regarding macrophages, Wang et al. [15] showed that only long MWCNT (0.6 mg/rat, corresponding to 3 mg/kg b.w.; length 20–50 μm and diameter 50 nm) stimulated the release of TGF-β in vivo, as measured by immunohistochemistry, contrary to short CNT (length 0.5-2 μm and diameter 50 nm). Long MWCNT (10 μg/ml; length 20 μm), but not short fibers (length 0.6 μm), were also shown to affect alveolar macrophage function, including cell death, ROS generation and pro-inflammatory (IL-6 and IL-8) mediator release [58]. A stronger ROS generation was also observed in A549 epithelial cells exposed to long CNT (length 5–30 μm; doses 5–20 μg/ml) compared to short fiber (length 0.5–2 μm) [76]. Poland et al. [3] reported frustrated phagocytosis in the peritoneal lavage of mice exposed to long MWCNT (50 μg/mouse, corresponding to 2.5 mg/kg b.w.), whereas short and tangled MWCNT were completely phagocytosed by macrophages. An accelerated rate of clearance of short CNT by macrophages has been reported, suggesting that these particles could be taken up by inflammatory cells leading to their translocation from the lung and, possibly, biodegradation, while long CNT seem to be biopersistent [77, 78]. Long MWCNT and SWCNT (length of 5–9 μm and 0.5–100 μm, respectively) have been shown to cause an impairment of airway epithelium barrier contrary to short particles. However, short MWCNT (length 0.6 μm) were shown to induce greater IL-6, IL-8 and MCP-1 levels than long MWCNT (length 20 μm) in TT1 and ATII cells [67].

CNT diameter also appeared critical since CNT with a thin diameter have been shown to stimulate fibroblast proliferation and to induce fibrosis compared to CNT with a thicker diameter [34].

The presence of residual metals from the synthesis of CNT has been shown to be associated to oxidative stress. Aldieri et al. [79] observed that only Fe-rich MWCNT were significantly cytotoxic and genotoxic in murine alveolar macrophages and induced a severe oxidative stress, contrary to the Fe-free MWCNT, suggesting that metals can be important factors in promoting CNT-induced toxicity. These data were further confirmed in another study, in which two types of SWCNT with different iron content, 26 wt. % and 0.23 wt. % (0.12–0.5 mg/ml), generated different amount of hydroxyl radicals in RAW 264.7 macrophages, confirming that iron can act as a catalyst of oxidative stress [80].

Finally, CNT dispersion was also found as another critical factor. Several evidences show that well dispersed CNT stimulate proliferation and collagen production by lung fibroblasts [29, 30], induce the release of IL-1β from macrophages [13, 35, 36, 43–47] and TGF-β from epithelial cells [36, 47], compared to not dispersed CNT. The extent and localization of CNT and inflammatory cells in the lung was also related to the particle dispersion and agglomeration state before administration. Agglomerated CNT form granulomatous inflammation and fibrosis in larger airways, while, in general, well-dispersed CNT are able to penetrate alveoli and interstitial space leading to alveolar fibrosis, as discussed in the section on fibroblasts [15, 28, 29, 81].

## Conclusion

**Fig. 4:**
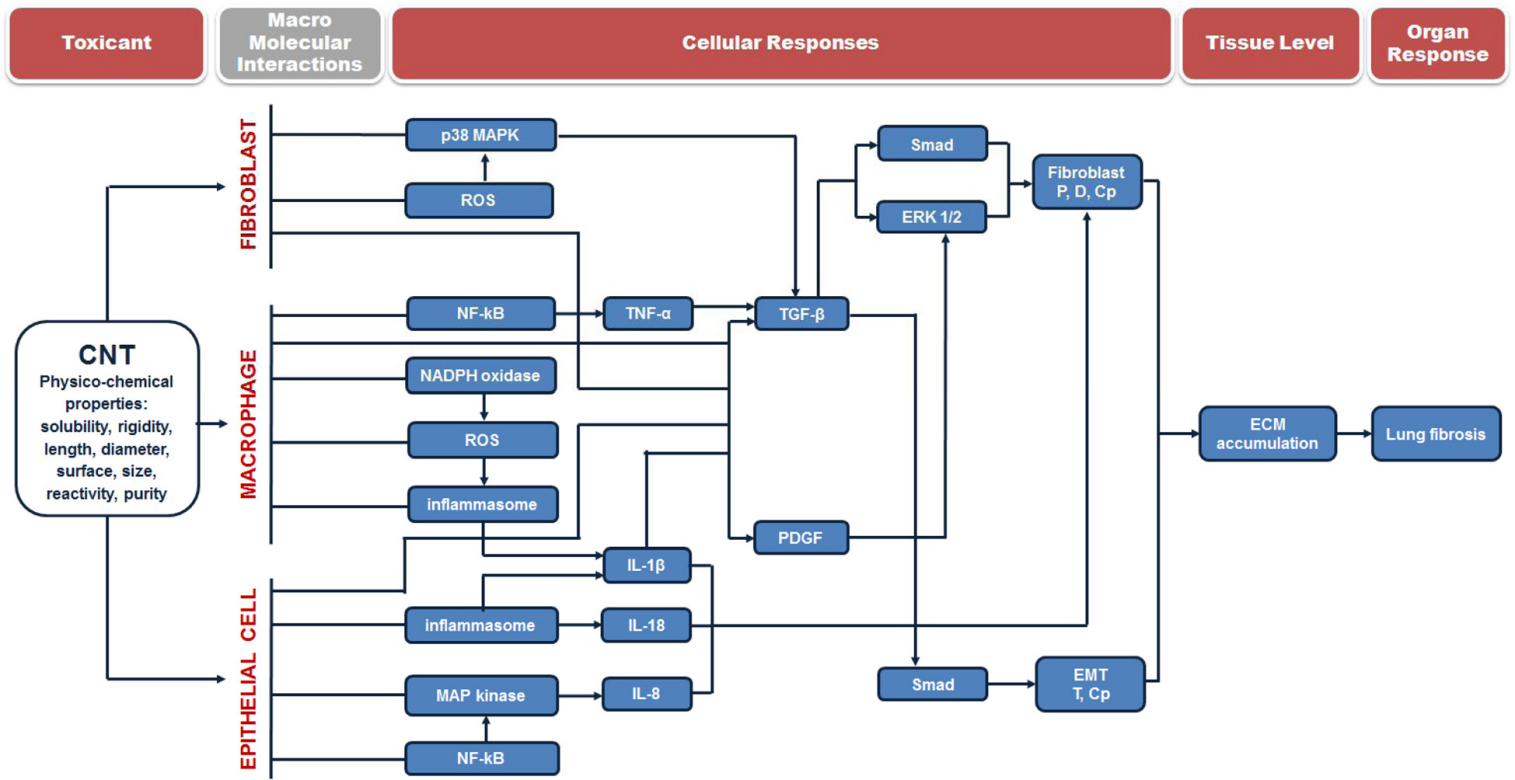
AOP for CNT and lung fibrosis. Legend: Cp: collagen production; D: differentiation; P: proliferation; T: transition in fibroblasts

The observations summarized in this review highlight indirect and direct effects of CNT on fibroblasts, which both contribute to their fibrogenic activity on the lung. In vitro studies indicate that a dose range of CNT between 1 and 100 μg/ml induced effects on the different cell types, as shown in Table 4, which summarizes in vitro data reported in this review. The dose range used in in vivo experiments (10–100 μg/mouse, corresponding to 0.5–5 mg/kg) is relevant for human exposure scenari, following the approach suggested by Porter et al. [82], and shown to exert a fibrotic activity in the lung of experimental animals. We propose in Fig. 4 a linear flow diagram based on an AOP structure describing the processes by which CNT have been shown to lead to fibroblast activation and pulmonary fibrosis. Physico-chemical properties of CNT, such as length and diameter are relevant for their fibrogenic activity. Therefore, an accurate analysis of these characteristics can already suggest whether a material may have or not a pro-fibrotic activity. The exact macro-molecular interactions (MMI) between CNT and cells have currently not been identified and we can only hypothesize that this probably occurs at the surface of membranes in fibroblasts, macrophages and epithelial cells. CNT have been observed to induce several cellular responses, which can be stratified according to 5 levels: i) initial signaling, ii) pro-inflammatory mediators, iii) pro-fibrotic mediators, iv) distal signaling in response to mediators and finally v) activation of key cells in fibrosis (fibroblasts and epithelial cells for EMT). ROS, MAPK, NF-kB, NADPH oxidase and inflammasome have been identified as the most important initial signals, with some differences depending on the cell type. Only macrophages and epithelial cells have been observed to release pro-inflammatory mediators with pro-fibrotic activities (IL-1β, IL-18, IL-8 and TNF-α) upon exposure to CNT. IL-1β seems to play a major role by promoting the secretion of pro-fibrotic mediators. PDGF and TGF-β can also be released directly from fibroblasts, macrophages or epithelial cells after CNT exposure and have been shown to activate the second signaling (ERK1/2 and Smad). Additionally, CNT prolonge ERK 1/2 activation in fibroblasts exposed to PDGF. ERK 1/2 and Smad phosphorylation was also prolonged by CNT in presence of TGF-β. ERK 1/2 and Smad signalings stimulated fibroblast proliferation, while Smad can also increase fibroblast differentiation, promote EMT and increase collagen production. This sequence of key events eventually causes collagen accumulation and ECM alteration and leads to the development of lung fibrosis.

**Table 4:** In vitro CNT activities on fibroblast, macrophage or epithelial cell

Cell		CNT					Concentrations	Effects	Mechanisms	References
Type	Name	Type	Source	Length (μm)	Diameter (nm)	Other				
Fibroblast	CRL1490	AP MWCNT D				BSA + DPPC		P		
		AP MWCNT ND				/		/		
		PD MWCNT D	CT	10–30	20–30	BSA + DPPC	5 μg/ml	P	nd	[30]
		PD MWCNT ND				/		/		
		COOH MWCNT D				BSA + DPPC		/		
		COOH MWCNT ND				/		/		
Fibroblast	MLg, HFL-1,	MWCNT NM400	N	0.7–3	5–35		7.5–30 μg/cm^2^	P		
	BALB-3 T3,	MWCNT NM402	A	0.7–4	6–20		(12–48 μg/ml)	P	nd	[23]
	PMF	MWCNT NM400c	N	0.1–0.5	18–35			/		
		MWCNTg 2400	PIH	0.7	20–50			/		
Fibroblast	MLg	MWCNT NM400	N	0.7–3	5–35		7.5–15 μg/cm^2^	P	KR, End, Erk 1/2	
		MWCNT long	CT	10–30	11–59		(12–24 μg/ml)	P	Erk 1/2	[34]
		MWCNT short	CT	0.5–2	10–47			/		
		MWCNT thick	CT	10–30	15–74			/		
Fibroblast	NIH-3 T3	MWCNT	NP	20–50	50		15–60 μg/ml	P, C, D	Smad	[24]
Fibroblast	CRL1490	SWCNT	CNI				0.02–0.24 μg/cm^2^	P, C, MMP-9	nd	[22]
							(0.03–0.4 μg/ml)			
Fibroblast	CRL1490	SD SWCNT	CNI	0.1–1	0.8–1.2	SD	0.02 μg/cm^2^	C	nd	[29]
		AD SWCNT				AD	(0.03 μg/ml)	C		
Fibroblast	CRL1490	SWCNT	CNI	0.1–1	0.8–1.2		5–25 μg/ml	P, C, TGF-β, VEGF	ROS, p38	[14]
Fibroblast	NHLF	Short SWCNT	CT	1	11		0.02–0.2 μg/cm^2^	C, TGF-β	ROS	[31]
		Long SWCNT		13	11		(0.03–0.4 μg/ml)	C, TGF-β		
Fibroblast	WI38-VA13	MWCNT	SA	2.5–20	6–13		1–20 μg/ml	cyt, TGF-β, PDGF	ROS	[13]
Fibroblast	L929	MWCNT	SA		5.5		10–300 μg/ml	cyt	ROS	[32]
Macrophage	THP-1	SWCNT	NI	1–5	0.8–1.2		50 μg/ml	IL-1β	IFM, ROS, End	[54]
		MWCNT	CT	10–30	20–30			IL-1β		
Macrophage	THP-1	SWCNT BSA-AP-Hipco	NI	1000–5000	0.7–1.1	BSA coated	50–100 μg/ml	IL-1β	IFM	[47]
		SWCNT BSA-PD-Hipco		500–1500	0.7–1.1	BSA coated		IL-1β	IFM	
		SWCNT PF108-Hipco		500–1500	0.7-1.1	PF108 coated		/		
		SWCNT BSA-AP-Arc		100–1000	1.2–2	BSA coated		IL-1β	nd	
		SWCNT BSA-PD-Arc		100–1000	1.2–2	BSA coated		IL-1β	nd	
		SWCNT PF108-Arc		100–1000	1.2–2	PF108 coated		/		
		SWCNT BSA-PD-SG65		450–2000	0.7–1	BSA coated		IL-1β	nd	
		SWCNT PF108-SG65		450-2000	0.7–1	PF108 coated		/		
Macrophage	THP-1	MWCNT	BNR		67		2–10 μg/ml	IL-1β	IFM, ROCK, End	[46]
Macrophage	THP-1	AP MWCNT D	CT			BSA + DPPC	10–100 μg/ml	IL-1β	nd	
		AP MWCNT ND	CT			/				
		PD MWCNT D	From AP	10–30	20–30	BSA + DPPC				[36]
		PD MWCNT ND	From AP			/				
		COOH MWCNT D	From AP			BSA + DPPC				
		COOH MWCNT ND	From AP			/				
Macrophage	PM, PBMC	short MWCNT	BMS	1–10	5–20			/		
		long tangled MWCNT	CT	10–50	8–15		10–100 μg/ml	/		[43]
		long needle-like MWCNT	MC	13	>50			IL-1β, IL-α	IFM, ROS, Src, Syc,	
		(Mitsui MWCNT-7)							P2X7 R	
Macrophage	PAM	raw MWCNT				/		IL-1β, IL-18	IFM	
	THP-1	pure MWCNT				PD		IL-1β, IL-18	IFM	
		raw MWCNT COOH	NAM			COOH F	25–100 μg/ml	/		[42]
		pure MWCNT COOH				COOH F - PD		/		
Macrophage	PHM	DWCNT	PIH	0.1–100	1.2–3.2		25 μg/ml	IL-1β, IL-18	IFM, End, K efflux	[44]
Macrophage	RAW 264.7	MWCNT	SA	2.5–20	6–13		2–20 μg/ml	IL-1β, TNF-α, IL-6, IL-10, MCP-1	NF-kB	[13]
Macrophage	THP-1, PBMC	MWCNT	HMS	0.5–40	10–30	Uncoated	10–100 μg/ml	IL-1β, TNF-α,	nd	[50]
						ALD-coated		IL-6, OPN		
								IL-1β		
Macrophage	PAM	Short MWCNT	Nth	1.1	30		10 μg/ml	/		[58]
		Long MWCNT		20	28			IL-6, IL-8	ROS	
Macrophage	THP-1	MWCNT AP	CT	0.57		F		IL-1β, TGF-β, PDGF		
		MWCNT NH2	From AP	0.45		F		IL-1β, TGF-β, PDGF		
		MWCNT sw-NH2	From AP	0.58		F		IL-1β, TGF-β, PDGF		
		MWCNT PEI	From AP	0.33		F	60 μg/ml	IL-1β, TGF-β, PDGF	for IL-1β: IFM, End	[45]
		MWCNT PEG	From AP	0.27		F		IL-1β, TGF-β		
		MWCNT cc-PEI	From AP	0.38		F		/		
		MWCNT COOH	From AP					/		
Macrophage	RAW 264.7	SWCNT	CNI		1–4		100 μg/ml	TGF-β	nd	[38]
Macrophage	RAW 264.7	Short MWCNT	NP	0.5–2	50		15 μg/ml	/		[15]
		Long MWCNT		20–50	50			TGF-β, TNF-α	nd	
Macrophage	NR8383	MWCNT	HMS	0.3–50	10–30		10 μg/cm^2^	PDGF	nd	[51]
							(16 μg/ml)			
Epithelial cell	A549	Short MWCNT	SN	0.35–0.7	10–20		1–16 μg/cm^2^	/		[65]
		Long MWCNT		5–15	10–20		(1.6–25.6 μg/ml)	EMT	TGF-β/Smad	
Epithelial cell	RLE-6TN	MWCNT	NP	20–50	50		30 μg/ml	EMT	TGF-β/Smad	[24]
Epithelial cell	HBE	MWCNT	HMS	0.5–40	10–30		1.5–24 μg/ml	IL-1β, IL-18	IFM, ROS	[16]
								IL-8		
Epithelial cell	BEAS-2B	MWCNT	BNR		67		1–10 μg/ml	IL-6, IL-8, MIF	NF-kB, p38,	[71]
									ERK 1/2, HSP27	
Epithelial cell	TT1, ATII	MWCNT 0.6 μm	Nth	1.1	30			IL-6, IL-8, MCP-1		
		MWCNT 3 μm		4.3	33		1–50 μg/ml	IL-8, MCP-1	JNK, p38, ERK 1/2	[67]
		MWCNT 20 μm		20	28			MCP-1		
Epithelial cell	BEAS-2B	SWCNT BSA-AP-Hipco	NI	1000–5000	0.7–1.1	BSA coated		TGF-β		
		SWCNT BSA-PD-Hipco		500–1500	0.7–1.1	BSA coated		TGF-β		
		SWCNT PF108-Hipco		500–1500	0.7–1.1	PF108 coated		/		
		SWCNT BSA-AP-Arc		100–1000	1.2–2	BSA coated	100 μg/ml	TGF-β	nd	[47]
		SWCNT BSA-PD-Arc		100–1000	1.2–2	BSA coated		TGF-β		
		SWCNT PF108-Arc		100–1000	1.2–2	PF108 coated		TGF-β		
		SWCNT BSA-PD-SG65		450–2000	0.7–1	BSA coated		TGF-β		
		SWCNT PF108-SG65		450–2000	0.7–1	PF108 coated		/		
Epithelial cell	BEAS-2B	AP MWCNT D	CT	10–30	20–30	BSA + DPPC	10–100 μg/ml	TGF-β		[36]
		AP MWCNT ND	CT			/		/		
		PD MWCNT D	From AP			BSA + DPPC		TGF-β		
		PD MWCNT ND	From AP			/		/	nd	
		COOH MWCNT D	From AP			BSA + DPPC		Few TGF-β		
		COOH MWCNT ND	From AP			/		Few TGF-β		
Epithelial cell	BEAS-2B	MWCNT	SA	2.5–20	6–13		2–200 μg/ml	TGF-β, PDGF	ROS	[13]

nd: not determined. *Cell name:* A549: human lung adenocarcinoma alveolar epithelial cell line; ATII: primary human alveolar type-II epithelial cells; BALB-3 T3: mouse embryonic fibroblast cell line; BEAS-2B: human bronchial epithelial cell line; CRL1490: human lung fibroblast cell line; HBE: primary human bronchial epithelial cells; HFL-1: human lung fibroblast cell line; L929: mouse fibroblast cell line; MLg: mouse lung fibroblast cell line; NHLF: normal human lung fibroblast cell line; NIH-3 T3: mouse embryonic fibroblast cell line; NR8383: rat alveolar macrophage cell line; PAM: primary alveolar macrophages; PBMC: peripherial blood mononuclear cells; PHM: primary human monocytes; PMF: primary mouse fibroblasts; RAW 264.7: mouse leukemic monocyte macrophage cell line; RLE-6TN: rat alveolar type II epithelial cell line; THP-1: human leukemia monocytic cell line; TT1: transformed human alveolar type-I-like epithelial cells; WI38-VA13: human normal lung fibroblast cell line immortalized with SV40. *CNT type:* AP: as prepared; COOH: carboxyl; NH2: amino; PD: purified. Source: A: Arkema (France); BMS: Bayer Material Science (Leverkusen, Germany); BNR: Bussan Nanotech Research (Ibaraki, Japan); CNI: Carbon Nanotechnologies Inc. (Houston, TX); CT: Cheap Tubes (Brattlebore, VT); HMS : Helix Materials Solution (Richardson, TX); MC: Mitsui & Company (Tokio, Japan); N: Nanocyl (Sambreville, Belgium); NAM: Nanostructured & Amorphous Materials Inc. (Houston, TX); NI: NanoIntegris (Skokie, IL); NP: Nanotech Port (Chengdu, China); Nth: Nanothinx (Rio Patras, Greece); PIH: produced in house; SA: Sigma-Aldrich (Lyon, France); SN: Shenzhen Nanoharbor (Shenzhen, China). *Other:* AD: acetone/sonication dispersed; BSA: bovine serum albumin; cc-PEI: carboxyl converted PEI; D: dispersed; DPPC: dipalmitoylphosphatidylcholine; F: functionalized; ND: no dispersed; PEG: polyethylene glycol; PEI: polyethyleneimine; PF108: Pluronic F108; SD: survanta dispersed; swNH2: sidewall amine. *Effects:* C: collagen; cyt: cytotoxic; D: differentiation of fibroblasts into myofibroblasts; EMT: epithelial mesenchymal transition; IL: interleukin; MCP: monocyte chemoattractant protein; MIF: migration inhibitory factor; MMP-9: matrix metallopeptidase 9; OPN: osteopontin; P: proliferation; PDGF: platelet derived growth factor; TGF: transforming growth factor; TNF: tumor necrosis factor; VEGF: vascular endothelial growth factor. *Mechanisms:* End: endocytosis; ERK 1/2: extracellular signal-regulated kinase; HSP27: heat shock protein 27; IFM: inflammasome; JNK: c-Jun N-terminal kinase; K: potassium; KR: kinase receptor; NF-kB: nuclear factor-kappa B; ROCK: Rho-kinases; ROS: reactive oxygen species

These data will be useful to guide researchers to focus on relevant mechanisms of action induced by CNT, or to investigate underlined knowledge gaps. The proposed AOP can help proposing or developing simple and fast test methods for predicting the potential hazard of CNT. Most importantly, it will help identifying how a mechanistic information obtained from one specific assay contributes to obtaining a global picture of the fibrogenic activity of a test sample.

## Abbreviations

A549: human lung adenocarcinoma epithelial cell line
AM: alveolar macrophages
AO: adverse outcome
AOP: adverse outcome pathway
ATII: primary human alveolar type-II epithelial cells
BEAS-2B: bronchial epithelial cells
BEAS-2B: human bronchial epithelial cells
BSA: bovine serum albumin
CB: carbon black
CNT: carbon nanotube
CRL-1490: human lung fibroblast
DCFH-DA: dichlorodihydrofluorescein diacetate
DHE: dihydroethidium
ECM: extracellular matrix
EGF: epidermal growth factor
EMT: epithelial-mesenchymal transition
EPR: electron spin resonance
FBS: foetal bovine serum
FESEM: field emission electron microscopy
FSP: fibroblast specific marker
GSH: glutathione
HBE: human bronchial epithelial cells
IL: interleukin
MIE: molecular initial event
MLg: mouse lung fibroblast
MMI: macro-molecular interactions
MMP: matrix metalloproteinases
MW: multi walled
NAC: N-acetyl cysteine
NADPH: nicotinamide adenine dinucleotide phosphate
N-cad: N-cadherin
NF-kB: nuclear factor-kappa B
NHLF: normal human lung fibroblasts
NIOSH: National Institute for Occupational Safety and Health
OPN: osteopontin
PDGF: platelet-derived growth factor
PMN: polymorphonuclear neutrophils
RAW 264.7: mouse leukaemic monocyte macrophage cell line
REL: reccomended exposure limit
RLE-6TN: rat alveolar type II cell line
ROS: reactive oxygen species
SMA: smooth muscle actin
SPC: surfactant protein C
SW: single walled
TBARS: thiobarbituric reactive substances
TEER: trans-epithelial electrical resistance
TGF: transforming growth factor
THP-1: human monocytic cell line
TIMP: tissue inhibitors of matrix metalloproteinase
TN: tenascin
TNF: tumor necrosis factor
TT1: transformed human alveolar type-I-like epithelial cells
VEGF: vascular endothelial growth factor
